# A Critical Review about Different Vaccines against Classical Swine Fever Virus and Their Repercussions in Endemic Regions

**DOI:** 10.3390/vaccines9020154

**Published:** 2021-02-15

**Authors:** Liani Coronado, Carmen L. Perera, Liliam Rios, María T. Frías, Lester J. Pérez

**Affiliations:** 1 National Centre for Animal and Plant Health (CENSA), OIE Collaborating Centre for Disaster Risk Reduction in Animal Health, San José de las Lajas 32700, Cuba; lcoronado@censa.edu.cu (L.C.); claura@censa.edu.cu (C.L.P.); mariat.frias@infomed.sld.cu (M.T.F.); 2 Reiman Cancer Research Laboratory, Faculty of Medicine, University of New Brunswick, Saint John, NB E2L 4L5, Canada; Liliam.rios@gmail.com; 3 Veterinary Diagnostic Laboratory, College of Veterinary Medicine, University of Illinois at Urbana–Champaign, Champaign, IL 61802, USA

**Keywords:** classical swine fever, vaccine, DIVA concept, genetic variability, multi-epitope vaccines

## Abstract

Classical swine fever (CSF) is, without any doubt, one of the most devasting viral infectious diseases affecting the members of Suidae family, which causes a severe impact on the global economy. The reemergence of CSF virus (CSFV) in several countries in America, Asia, and sporadic outbreaks in Europe, sheds light about the serious concern that a potential global reemergence of this disease represents. The negative aspects related with the application of mass stamping out policies, including elevated costs and ethical issues, point out vaccination as the main control measure against future outbreaks. Hence, it is imperative for the scientific community to continue with the active investigations for more effective vaccines against CSFV. The current review pursues to gather all the available information about the vaccines in use or under developing stages against CSFV. From the perspective concerning the evolutionary viral process, this review also discusses the current problematic in CSF-endemic countries.

## 1. Introduction

Classical swine fever (CSF) is one of the most devasting viral infectious diseases affecting the members of *Suidae* family [1,2]. The causative agent is a highly contagious, small, enveloped, single-stranded RNA virus known as CSF virus (CSFV), which together with other 11 viral species comprise the *Pestivirus* genus of the family *Flaviviridae* [3]. Since CSF-outbreaks have negative social and economic implications, including serious restrictions on international trade of pigs and their products, high financial burden due to direct or indirect loses in the pig industry, and the ethical aspects linked to the removal of herds in affected farms, it is considered a notifiable pathogen by the World Organization for Animal Health (OIE) [4]. Although some countries, such as the United States, Canada, Australia, and New Zealand, have successfully eradicated the disease, CSF remains endemic in most countries in Asia, Eastern Europe as well as South and Central America, and the Caribbean. In the European Union, despite the status-of-freedom for CSF, there is a constant threat, since the virus remains endemic in wild boar populations, causing sporadic outbreaks by its re-introduction into domestic herds in some Member States with large swine populations [5,6]. Similarly, the reemergence of CSFV in several countries in America, including Ecuador [7], Brazil [8], and Colombia [9], as well as in Asia, such as Japan [10] and South Korea [11], sheds light about the serious concern that a potential global reemergence of this disease represents.

It is relevant to denote that in the past, most of the countries that achieved the *free-status* of CSF used stamping-out policies, for instance, Australia in 1961 combined stamping-out policies with movement restrictions [12]; Canada in 1963 reached the eradication of CSF by combining vaccination/stamping-out policies; the United States used stamping-out policy in 1976 after suspending the vaccination in 1962 [13]; and the EU, after 10 years of pursuing the ambitious plan of eradication under the EU Council Directive 80/217/ECC, reached the status of Free-CSF with its last Member State, Italy, in 1990 [14]. This policy of massive number of animals subject to welfare slaughter may be impractical for the existing pig farms in the current days because of the elevated cost and unethical aspects behind this method [6]. The negative aspects about the application of mass stamping out policies gain strength if it is also considered that several countries have also achieved the status of CSF-free using vaccination as main control measure, including Uruguay in 1991 [15], Chile in 1998 [15], and Argentina in 2005, sustaining the fact that the massive slaughtering of swine herds (mainly those neighboring unaffected herds) are policies from the past and incoherent with the current status of the science, regulatory laws, and rationality of the societies. Nonetheless, it is also worthy to clarify that the vaccination process executed under poor control measures, without a proper vaccine dose or failing to establish a sterilizing immunity in pig herds, could lead to disastrous consequences like the emergence of escaping variants of CSFV strains [16] and reemergence of the diseases [10], among others [17]. It is also important to highlight the role played by the different diagnostic methods in the eradication process of CSF. In fact, the proper detection of infected animals is a key factor for the accurate segregation of the animal and the control of the disease [18,19]. In this regard, a successful eradication program must include, among other factors, the creation of diagnostic laboratories with the capacity to properly perform molecular diagnostic techniques as part of the routine methods of diagnostic for the accurate and reliable detection of CSF [19]. Likewise, the inclusion of detailed studies about the phylogenetic links between the circulating viral strains with the subsequent characterization of the pathogenesis has been suggested as essential steps to guarantee efficient intervention strategies for the control of CSFV [16,20]. However, all these aspects require the intervention of governmental policies to ensure the proper funding, organization and execution of the control programs, but a relevant starting point is the use of an efficient vaccine.

Hence, it is imperative for the scientific community to continue with the active investigations for more effective vaccines against CSFV. There is an urgent need for effective products (novel vaccines) that in conjunction with additional control measures can be applied in a rational way to control or mitigate the losses caused by outbreaks of CSFV. Therefore, the current review pursues to gather all the available information about the vaccines in use or in developing stages against CSFV (see Table 1). We also focused on the current problems (from an evolutionary viral process point of view) faced by CSF-endemic countries. Thus, the main aim of the current review is to provide summarized information that can be used by research groups to acquire a clear understanding about the *status quo* regarding the vaccination against CSFV with emphasis in its repercussion on endemic areas.

## 2. Live Attenuated Vaccines

Historically, massive vaccination using live attenuated vaccines have been implemented in several countries as a mandatory control program for more than 50 years [6,21,22], which together with additional biosafety measures has successfully conducted to the eradication of CSF [21,22]. These vaccines contain as main immunogen strains of CSFV [23], which have been attenuated by adaptive mutations obtained through serial passages in either rabbits or cell culture. Among the most commonly used strains are the Lapinized Philippines Coronel (LPC) strain, the C-Strain or so-called “Chinese hog cholera lapinized virus” (HCLV), Russian vaccine strain LK-VNIVViM, the low-temperature adapted Japanese guinea-pig exaltation-negative (GPE-) strain, the French cell culture adapted Thiverval strain, and Mexican PAV strains [24,25,26]. This type of vaccine presents several properties that facilitate its use, highlighting their low cost and its straightforward manufacturing. The CSFV live attenuated vaccines have shown to be safe to the target animals including the two principal categories: young pigs and pregnant sows [22], which is the main characteristic of these type of vaccine. In addition, several studies have shown the capacity of the life attenuated vaccines to provide early protection against virulent strains even at 1 day post-vaccination and the administration of a single dose. [27,28,29,30]. Another claimed advantage of this type of vaccine relies in its capacity to elicit both cellular and humoral immune responses, providing sterilizing protection in pigs. Results that support the previous statement have proven, in fact, the production of CSF virus-specific gamma interferon as early as 6 days post vaccination (dpv), which has been linked as a key component of the cellular immune response [27,31,32,33]. The immunity provided by the life attenuated vaccines can last from 10 months, if administered orally [34], to lifelong CSF immunity with a single intramuscular inoculation [24,35]. In addition to horizontal protection, this type of vaccine induces vertical protection in pregnant sows as early as 5 dpv, preventing the viral transplacental transmission [21,22]. Nevertheless, for some years, there has been a strong controversy about the advisability of using this type of vaccine. In the EU, where prophylactic vaccination is banned, emergency vaccination can be implemented in cases of severe outbreaks in domestic pigs [36]. However, current live attenuated vaccines against CSF elicit the full spectrum of antibodies, and vaccinated pigs cannot be distinguished from infected pigs by serological methods. Therefore, the use of live vaccine is followed by severe trade restrictions [37]. The availability of safe and effective DIVA (Differentiating Infected from Vaccinated Animals) vaccines and a corresponding laboratory diagnostic test could solve this dilemma if DIVA vaccination becomes an internationally accepted method for emergency vaccination without disruption of trade.

The abovementioned attenuated vaccines have been used for control and eradication of CSF in several regions of the world [38]. These vaccines have common characteristics in terms of genetic stability, protective immune response, but also have certain differences. These differences are mainly based on their capacity to replicate into the vaccinated animals, the timeframe of the viremia, the route, and capacity of excretion of the viral vaccine strains, among others.

### 2.1. LOM Vaccine

The LOM strain was initially derived from a low virulence strain of Miyagi isolate from Japan in 1956 and further attenuated through continuous propagation in bovine kidney cell culture [39,40]. LOM strain was first tested as a vaccine candidate from 1968 to 1969 in the field by the Institute of Veterinary Research (IVR) in South Korea [41]. Subsequently, this live attenuated vaccine came to be widely used throughout the country to eradicate CSFV since 1974. The fact that LOM vaccine has been used in South Korea during so many years, indicated that this vaccine is presumably safe with a high immunogenic response in pigs for many decades, however, sporadic outbreaks of CSF have occurred continuously [42,43,44]. In addition, it is important to denote that the LOM strain has been kept through several passages in bovine/porcine kidney cells for many years, and despite this strain has been considered genetically stable, there have been hints of a potential reversion to a virulent strain [45]. Indeed, after the unintentional vaccination of the LOM vaccine strain in 2014, CSFV reemerged in Jeju Island, South Korea, which had been a CSF-free region with a non-vaccination policy for a decade [46]. Since the reemergence, endemic outbreaks of CSFV have occurred in the island, causing enormous damage to pig farms [47,48].

### 2.2. C-Strain Lapinized Vaccine

The C-strain vaccine has been regarded as one of the most effective CSF vaccines used worldwide for the control of CSF in domestic pigs. The origin of this strain is unclear [49] but it has been reported that the C-strain was developed by the Chinese Institute of Veterinary Drugs Control and the Harbin Veterinary Research Institute in 1956 [38,50]. Several vaccines have been developed from C-strain of CSFV in different countries, such as Pestiffa in France, SUVAC in Hungary, Cellpest in Poland, Suiferin C in former East Germany, VADIMUN in USA, Duvaxin and Riems in Germany, Norden and Porcivac in Mexico, PS Poreo in Brazil, Tipest in Slovakia, TVM-1 in Czech Republic, and Russian LK in Russia [25,37].

Lapinized vaccines are widely acclaimed as highly safe and effective against the disease, which also can elicit protective immune response against all CSFV genotypes [24,28,39,51,52]. However, a recent study warned about the lack of capacity of the lapinized vaccines to protect against all genotypes reporting the emergence of neutralization-escape mutants from the genotype 2 of CSFV strains in China [43] Although the authors claimed a reduced neutralization efficiency for anti-C-strain polyclonal antibodies and suggested the need to develop a new CSFV vaccine based on genotype 2 to prevent this vaccine-escaping mutants [43], an additional report [53] pointed out the lack of evidences to support the claims made by Yoo et al. [43]. Rios and Perez [53] evidenced that Yoo et al. [43] failed to conduct specific experiments using monoclonal or polyclonal antibodies, which would have provided the necessary information to claim that these mutants were indeed neutralization escape mutants [53], among others aspects deeply analyzed in [53]. Therefore, based on the current evidences, it is still widely considered that the lapinized vaccines have broad protection against all CSFV-genotypes. 

An essential feature of the C-strain vaccines is their capacity to induce protection even a few days after immunization and prior to seroconversion [27]. This early protection has been shown to rely upon the induction of cell-mediated immunity [31]. In fact, CSFV-specific gamma interferon (IFNγ) secreting cells has been detected in pigs vaccinated with C-strain, as early as 6 dpv [32,34,54]. On the other hand, the onset of protection following C-strain vaccination relies on the administration method [22]. The establishment of complete clinical protection against the virulent strains after oral immunization at 10 dpv has been shown by a previous study [34]. A recent study also revealed that the vaccination using C-strain stimulates the proliferation of T-helper cells (Th-cells) [55]. It is well known that the subpopulation of Th2 cells secrete IL-4, IL-5, IL-6, IL-8, IL-10, and IL-13, which play a relevant role in the direction of the immune response stimulating B cell proliferation, inducing B cell differentiation that end up to antibody class switching, as well as improving the neutralizing antibody production [55]. These authors also exposed that the vaccination using C-strain vaccine was not linked to an increase in the expression levels for TNF-a or IL-1 and suggested, by this mean, that the vaccination using this type of strains can not cause any damage as a result of inflammatory response. Additionally, it was also revealed that the IL-6 levels started to increase at 7 dpv, with a maximum level of expression at 16 dpv with a 1000-fold upregulation, and decreasing thereafter, which suggests an important role in immune regulation after vaccination with the C-strain vaccine [55]. An additional advantage of the C-strain vaccines is their slow and limited replication (to small viral titers) in tissues, this aspect could favor the temporary escape from the recognition of Toll-like receptors (TLRs) avoiding the early overexpression of IFNs and pro-inflammatory cytokines protecting the tissues and organs indirectly [55].

Furthermore, the immunity conferred by C-strains has been widely accepted as long-lasting, persisting for a lapse of time from 6 to 11 months or even lifelong [22,36,37,56]. Vaccination using C-strains has proved to confer full clinical protection (vaccinated animals do not show clinical signs compatible with CSF) and sterile protection (lack of viremia, absence of viral shedding, and not viral particles or genome detectable) [57]. Besides the remarkable efficacy, C-strain vaccines have shown to be highly safe in both, target and non-target species [51] with the major advantage that the transplacental infection by the virulent viral strains is prevented [52]. Moreover, several studies have shown that the lapinized vaccines or C-strain vaccine do not reverse their virulence, even after 30 passages in pigs [23]. Hence, this type of vaccine is still one the most suitable vaccines to use for control purposes mainly in underdeveloped countries since besides the high level of efficacy and safety, the production of these vaccines is technologically easy to perform, cost-effective, and do not require adjuvants and they are suitable for oral vaccination of wild boar populations [58]. 

### 2.3. GPE-Strain

The Japanese guinea-pig exaltation-negative strain (GPE-) vaccine was developed in 1969 by the Department of Exotic Diseases at the National Institute of Animal Health in Tokyo, Japan. This attenuated vaccine strain was derived from the wild-type ALD strain through multiple passages and biological cloning in swine testicle cells, bovine testicle cells, and primary guinea pig kidney cells [54]. Pigs inoculated with the GPE- vaccine did not develop such clinical symptoms as anorexia and pyrexia, the GPE- strain rarely produced viremia in the inoculated animals and did not show evidences of shedding in excretions [56]. Moreover, the vaccination based on GPE- strain has shown to be safe in pregnant sows, newborns, and adult pigs. Likewise, this live attenuated vaccine confer protective immunity against the development of the clinical signs compatible with CSF as well the viral replication and dissemination [54]. Initial reports observed the protection capacity of GPE- vaccine as early as 3 dpv [59]. The humoral response characterized by the presence of neutralizing antibodies in vaccinated pigs has shown to start between 10 and 14 dpv lasting for at least 2 years without a reduction in antibody titers observed [54]. A recent study warned the scientific community about the capacity of the viral strain GPE- to revert to the virulence after 11 serial passages in pigs [60]. The reversion to a virulent strain of GPE- was linked to the substitutions of three amino acids, T830A in the E2 protein and V2475A and A2563V in the NS4B protein, [60]. Nonetheless it is important to denote that despite the risk that this novel discovery arise the vaccines based on the Japanese GPE- strain have been used in Asian and Pacific countries for decades [37].

### 2.4. Thiverval Strain

The Thiverval vaccine strain was derived from virulent CSFV strain Alfort through more than 170 serial passages in cells at 29–30 °C. This vaccine was patented in France approximately in 1971, had shown genetic stability and high degree of safety even when was applied on immunosuppressed animals [61]. In addition, Thiverval vaccine strain did not show a residual virulence or reversion to virulence factors [62]. During many decades of collected evidences Thiverval vaccine has shown high level of efficacy, safety as well as the capacity to prevent vertical transmission of CSFV when used in pregnant sows. Likewise, the vaccination using Thiverval strain did not have effect on the development of gestation or on newborn fetuses [61]. 

### 2.5. PAV-250

The PAV-250 strain was obtained from CSFV strain A attenuated by 250 sequential passes in the PK15 cell line. This live attenuated vaccine has been successfully used in the program for the control and eradication of CSF in Mexico since 1979 [63]. PAV-250 has been widely tested on pigs, conferring both clinical and antiviral protection, with an immunological response started between 3 and 5 dpv. Like other live attenuated vaccines PAV-250 has been shown to be safe in pregnant sows as well in other categories. In addition to the antigenic capacity of PAV-250 to elicit a strong immunogenic response, this vaccine has shown a high genetic stability, a lack of transmissibility, and the capacity to protect against different virulent strains of CSFV [64,65]. PAV-250 vaccine has also shown no signs of reversion to virulence and have the capacity to persists in the tonsils up to 28 dpv with a short time of viremia up to 7 dpv, determined by RT-PCR technique [64].

In general, live attenuated vaccines against CSFV have several advantages, including safety, broad range of protection but a major disadvantage of this type of vaccines is that they are unable to discriminate between infected and vaccinated animals based on their serological profiles. Therefore, this vaccination strategy do not exempt of imposing severe trade restrictions to those countries that apply live attenuated vaccines to control CSF under an emergency vaccination scenario [6]. Hence, in order to sort out this issue, several research groups around the world are in the unflagging search of novel vaccines candidates against CSFV with promising results in this area. 

## 3. Marker Vaccines

The concept of marker vaccines arises from the need to differentiate infected animals from those vaccinated ones. Associated with the DIVA concept explained above, each marker vaccine must be coupled with a discriminatory test, which must be able to selectively determine which vaccinated herds are or not free to circulating field strains [25,66]. In addition to the main purpose for which this type of vaccines emerged, they are considered relevant tool in fundamental investigations to uncover the mechanisms involved in the induction and control of immune response. In this regard, several investigations have been recently conducted to elucidate the mechanisms involved in the induction and control of the humoral and cellular immunity with the subsequent characterization of T and B epitopes in conjunction with the genomic analysis and the emergence of the immunoinformatic as a scientific discipline [16,32,67,68,69,70,71]. In general, the development and manufacturing of the marker vaccines have covered, so far, four main strategies, including, subunit vaccines, viral vectors (chimera vaccines and replicons), immunogenic CSFV peptides, and DNA vaccines [39,72,73].

### 3.1. E2 Subunit Vaccine

A popularized option of marker vaccines emerged using the E2-protein, which is considered the most immunogenic viral protein of CSFV in non-replicating systems [24]. This first generation of genetically engineered vaccines is recognized in the scientific literature as E2-subunit vaccine anti CSFV [72,74,75,76,77]. Analyzing in detail, E2-protein is the major virus structural glycoprotein, is considered an essential component of the viral envelope and play a relevant role during the viral infection since it is responsible together with E1-protein in the viral attachment to the cellular receptor [78]. E2 is highly immunogenic and it is linked to the induction of neutralizing antibodies, which have a protective role against the viral infection [73]. These elements pointed out that the glycoprotein E2 is an ideal candidate for the development of different strategies of recombinant vaccines against CSFV [75,77,79,80,81,82,83,84].

Based on the described characteristics of E2, the whole protein or regions from the main epitopes of E2, have been used coupled with different expression systems to generate several commercial vaccine candidates. The first commercial subunit vaccines were launched after several investigation using the system of baculovirus-expressed E2 protein in insect cell line [72,74,75,77,83]. One of the major advantages claimed by baculovirus-expression system was the capacity to resemble the natural glycosylation pattern of E2, which is a critical factor for activation of the immune system [85]. Indeed, the E2-expressed protein into baculovirus-system with a subsequent adjuvant using a water–oil–water emulsion, showed to be able to induce protection against CSFV virulent strains [86]. Thus, two E2-subunit vaccines were authorized by the European Medicines Agency (EMEA). One from Bayer, Leverkusen, Germany, Bayovac^®^ CSF Marker (Bayer, Leverkusen, Germany), and the other one from Intervet, Boxmeer, The Netherlands Porcilis^®^ pesti (Intervet International BV, Boxmeer, The Netherlands). However, further investigations performed downstream, showed numerous issues with these licensed vaccines. In fact, after numerous conducted vaccination-challenge experiments, the high level of safety was verified regarding these types of vaccines [87,88,89,90,91,92,93], however, issues related with the late onset of immunity and the protection conferred with subunit vaccines were pointed out, revealing their limited capacity. 

A major concern was raised after the revelation of the incapacity of these vaccines to induce vertical protection and, the high risk of establishment of persistently infected pigs prompted by the incapacity to prevent the transplacental transmission of CSFV in pregnant sows [22,25,91,92,94,95]. As an additional problem, it was revealed that in order to provide a sterilizing protection, to prevent the horizontal transmission of CSFV, this type of vaccines requires two vaccinations doses via interparental [22,86,96]. Despite the needed two doses the licensed E2 marker vaccines induced a shorter immunity compared to the live attenuated vaccine lasting approximately only from 6 to 13 months [88,90,91,97]. As major disadvantage was also pointed out the fact that since these vaccines do not replicate into the host, they were ineligible for oral administration making them unsuitable for the oral vaccination programs, targeting the endemically infected wild boar populations of relevant importance during emergency vaccination campaigns [2,97]. Based on the mentioned inconvenience in the current days, only Porcilis^®^Pesti (Bayer, Leverkusen, Germany) (containing the E2 glycoprotein of CSFV strain Alfort-T) is available in the market [37].

Thus, to overcome these drawbacks, several research groups have focused on the development of novel candidates to fulfil either general requirements including safety, effective clinical protection, prevention of horizontal and vertical transmission or in particular requirements such as the induction of a specific antibody response [21,98]. In this regard, a more recent vaccine candidate based on baculovirus-expressed E2-system has shown an sterilizing protection after a single vaccination dose [83]. A subsequent study from this same group of research got deep insight into the minimal requirements of the E2 antigen concentration to confer rapid and long-lasting clinical protection against CSFV as a key element of this novel candidate called “KNB-E2” [94].

This same year, in 2018, a third E2-subunit vaccine candidate called “Tian Wen Jing, TWJ-E2^®^” was officially licensed in China, with its further commercialization. This vaccine also was implemented based on the well-known baculovirus-expressed E2 system with a moderated modification using as antigen the E2 glycoprotein of a subgenotype 1.1 C-strain [84]. The TWJ-E2 vaccine has shown to provide full protection against a challenge using the highly virulent genotype 1.1 reference strain *Shimen* after two doses. Likewise, vaccinated pigs with TWJ-E2 showed elevated titers of neutralizing antibodies against E2 and were fully protected against a lethal challenge by CSFV field strains from two different genotypes 1 and 2 [84]. However, a subsequent study revealed that the stimulation of the cellular immunity was still insufficient, which seems to be linked to the fact that the onset for a solid immunity against CSFV depends upon two-doses of the vaccine, and it is achieved at 14 dpv [81]. This issue denotes that this novel TWJ-E2 vaccine does not have a clear advantage with the previous commercialized candidates. Moreover, it remains unclear whether this new licensed TWJ-E2 vaccine has the capacity to provide sterilizing immunity against field CSFV-strains in pregnant sows, therefore, its role of protection against the vertical transmission of CSFV is still a concerning question. 

Taking into consideration fundamental knowledge emerged from molecular immunology, recent strategies have been focused on increasing the capacity of the subunit vaccines, targeting the cellular immune response and boosting the humoral immune response at the same time [37]. Thus, different research groups are looking for novel molecular adjuvants, which can induce a broader cytokines response with the subsequent stimulation of particular subpopulations involved in the activation of the cellular response [76,99,100]. Examples regarding this novel strategy seems to provide promissory results, for instance, a new CSF subunit marker vaccine based on the combination of the E2 protein in fusion with the extracellular domains of porcine CD154 has been recently developed [95,96]. The CD154 molecule is a member of glycoproteins that integrate the tumor necrosis factor (TNF) superfamily, and it has been defined as the most important co-stimulator of activating antigen-presenting cells [101,102]. In addition, the CD154 receptor (CD40) belongs to the same TNF superfamily located at the surface of B cells, dendritic cells (DCs), macrophages, Langerhans cells, epithelial cells, endothelial cells, and fibroblasts [103]. During the activation of the humoral immune response, CD154-CD40 signaling plays crucial roles during the proliferation and differentiation of antigen-responding B cells, antibody isotype switching as well as affinity maturation. All these processes are essential for the generation of memory B cells and long-lived plasma cells [99,104]. Furthermore, CD40 signaling is critical for the expansion and differentiation of antigen-specific T cells and can influence T cell-mediated effector functions [103]. In fact, it has been demonstrated that the disruption of the CD40/CD154 pathway results in poor CD4+ T helper cell proliferation in response to antigen exposure, inhibition of IL-4, and IFNγ production and failure to generate antigen-specific T cell responses [100,104]. Considering the capacity of CD154 to activate subpopulations linked to elicit humoral and cellular immune responses, this immunomodulator have been suggested as a key adjuvant that could improve the deficiencies previously found in the subunit vaccines [105]. Experiences accumulated from other vaccine candidates against other viral species such as influenza A virus also supported the previous statement [99,105]. The studies conducted on the novel candidate E2-CD154 subunit vaccine have revealed the ability of this candidate to fully protect pigs from very virulent strains of CSFV as earlier as 7 dpv after a single vaccine dose, which resulted in a significant advance compared with previous candidates or licensed E2-subunit vaccines [96]. This novel candidate has shown the capacity to elicit full protection against the challenge of very virulent strains of CSFV in the absence of protective neutralizing antibodies, which seems to be indicative of an increased number of CSFV-specific IFNγ producing cells in animals vaccinated with E2-CD154 vaccine [96,106]. Based upon the results obtained from the controlled experiments, there are evidences that this novel candidate could confer an early protection against CSFV like the one provided by the live attenuated vaccines. Furthermore, the E2-CD154 subunit vaccine candidate showed to prevent against the vertical transmission of the very virulent CSFV strain in pregnant sows representing a major advantage for this type of candidates, and so far, is the only subunit vaccine showing this feature as a significant remark. [95]. Additional results from the evaluation regarding the stability of the E2-CD154 subunit vaccine showed that this novel candidate was able to keep its biological properties including potency at least during 24 months after being storage at room temperature [107]. This is a remarkable aspect to consider, taking into account the fact that CSF is endemic mainly in tropical areas specially in underdeveloped countries, where ensuring the mandatory cold-chain to preserve the potency of the live attenuate vaccines is a significant concern [6].

Engaging with the fundamental principle, “from the laboratory to the field” the E2-CD154 vaccine has been already tested on different pig farms under CSF-endemic conditions, using Cuban swine herds as scenario for this purpose [16,20]. Thus, under these uncontrolled conditions, elevated titers of neutralizing antibodies against CSFV were detected, which lasted for at least 9 months, however, it is important to highlight that in these cases, two doses at 3 weeks after the first vaccination was conducted [107]. Considering the promising results presented by this candidate, this research group continues to conduct additional studies to fulfill all the requirements established by the OIE for candidate vaccines, including safety and efficacy.

On the other hand, it is well known that the use of cytokines could be an attractive choice to develop subunit vaccine formulations with the aim to induce an early protection against CSFV. During the last decade, Toledo et al. [108] proposed the use of alpha interferon (IFNα) to increase the immunogenicity of a vaccine candidate based on the E2-CSFV antigen produced in goat milk (INFα-E2-CSFV) [108]. The IFNs type I are components from the innate immunity, which modulate a number of molecular pathways to activate the acquired immunity by synergic mechanisms that are critical for the activation of the antigen-specific immunity also known as adaptive immune response [109]. For instance, during the development of the humoral immune response, type I INFs enhance the primary antibody response against soluble proteins, stimulating all IgG subclasses as well as the subpopulations involved in the response of the immunological memory [110]. In addition, Type I IFNs play a role in the maturation of dendritic cells and act as survival factor for activated T cells [110]. In the study presented by Toledo et al. [108], the authors evidenced that INFα used as adjuvant in the formulation together with E2-CSFV antigen boosted a specific response of anti-CSFV-neutralizing antibodies, showing higher antibodies titer than in immunized animals with E2-CSFV alone. Besides, CSFV-specific neutralizing antibodies were not detected in any of the pigs of the group immunized with E2-CSFV/hαIFN co-formulation as early as 7 dpv, however, at this time, these animals were protected against clinical signs and sign of viremia [108]. This fact demonstrates that the protection conferred by this co-formulation is independent of the humoral immune response. Thus, during this early stage, protection seems to be determined by the antiviral activity of IFN and probably by a cellular immune response, also enhanced by the IFN. Moreover, the induction of an early, and long-lasting protection against the challenge with CSFV virulent strain after a single dose of this vaccine was also observed by this research group [111]. Based on this approach of using IFNs as immunoadjuvant, Zhang et al. proposed a new subunit vaccine co-expressing the E2-protein of CSFV together with porcine IFNγ in a baculovirus expression system [112]. It is important to denote that IFNγ is the only member of the type II IFN family, it is mainly produced by activated T cells, and it is linked to the differentiation from Th0 to Th1 population, the activation of natural killer cells and macrophages, enabling the protection against CSFV [113]. In fact, in the study presented this research groupthe novel candidate did not enhance the CSFV-specific antibody and neutralizing antibody titers compared with the E2 subunit vaccine alone but significantly enhanced the CSFV-specific IFNγ expression with a subsequent increase in the cellular immune response specific against CSFV. This novel candidate also conferred complete protection against CSFV, hence, it has been proposed by this research group as a promising marker vaccine candidate for the control and eradication of CSF [112]. 

One of the major attempts of the current review is to standardize the advantages and disadvantages of the different vaccination strategies, whereas this is possible across different vaccine types, the comparison among subunit vaccines, in specific, is difficult since they are quite diverse in formulations, have different inoculation routes, etc. Nevertheless, from a general point of view, subunit vaccines have several aspects in common. In brief, subunit vaccines have a major advantage over live attenuated vaccines regarding safety, since this type of vaccine are non-replicating viral particles, there is little possibility of reversion to virulence. Furthermore, this type of vaccine has shown greater thermal stability than live attenuated vaccines favoring its use in underdeveloped countries. A clear weakness shown by most of the candidates published by different research groups has been their poor or lack of induction of cellular immune response and shorter duration of immunity than the live attenuated vaccines. As a direct consequence of this last characteristic, the subunit vaccines may require intermittent immunization or two doses to increase antibody titers in order to maintain active immunity to the chosen antigen. Besides, multiple doses, using a considerable concentration of antigen will definitely increase the vaccination costs, causing extra economic burden to the pig production [114]. 

Although marker E2 sub-unit vaccines have the potential for differentiation, they lack some critical properties offered by live vaccines, such as rapid and complete protection after a single application, prevention of transplacental infection, and suitability for oral use. With the purpose of developing DIVA vaccines, which at the same time evoke both cellular and humoral immunity, other different approaches have been pursued in the last years. 

### 3.2. Live Marker Vaccine

With the advent of the reverse genetic technology, new strategies have emerged to support the rational design of novel vaccines against CSFV, which could gather both major advantages of the vaccination strategies discussed above, the ability to induce the cellular immune response observed from the live attenuated vaccines [27] and the DIVA principle from the subunit vaccines [25]. In this direction, a novel generation of engineered vaccines has emerged with a promising perspective and recognized in the scientific community as live marker vaccines [37]. Within this group, the construction of chimeric pestivirus and replicons have been already tested and accumulated results for most than 10 years. 

## 4. Chimeric Pestiviruses

Chimeric pestivirus is a new concept based on the close relationship between pestivirus, which provides new options for designing improved CSF marker vaccine candidates. These genetics constructions are considered the most promising second-generation candidates to develop CSFV DIVA vaccines with the potential to combine the efficacy of live attenuated vaccines with marker properties [39,73,115,116]. Recently, CP_E2alf vaccine, was licensed as the first live marker vaccine against CSF, produced under the name ‘‘Suvaxyn^®^ CSF Marker” by Zoetis and approved by EU for emergency vaccination within restricted control [117]. The implementation of an emergency vaccination program using a marker vaccine is aimed to avoid the ethically, questionable, and expensive “stamping-out” strategy, which increases the public acceptance of the eradication policy and is cost effective.

The chimeric pestivirus marker vaccine CP7_E2alf was developed using the CP7 bovine viral diarrhea viral strain (BVDV) backbone that expresses the E1 and E2 glycoproteins of the CSFV strain Alfort/187 [118,119,120]. Several studies about this genetically engineered virus proved the efficacy and safety of this DIVA vaccine candidate after intramuscular vaccination of domestic pigs and wild boar populations with the additional feature that enables its use for oral administration in [115,116,118,121,122,123,124,125]. The results obtained using Suvaxyn has demonstrated either clinical and virologic protection after the challenge against virulent and moderate CSFV strain [126,127,128]. This product has also sorted out the effect that the presence of maternal-derived antibodies could have in the development of an immune response to the vaccination [124,129]. In addition, it has been extensively documented that the use of Suvaxyn confers complete protections against the virulent strains of CSFV after a week of applying a single intramuscular dose, and the immune protection can last for at least 6 months [126]. Initial results focused on to elucidate the protection capacity of CP7_E2alf vaccine against the vertical transmission was not undoubtedly shown, especially with early and harsh challenge infection [90]. However, a recent study showed that pregnant sows and their fetuses were fully protected after a single dose of this DIVA vaccine, but it is important to denote that in the mentioned study, the challenge was conducted using a CSFV strain of moderate virulence [130]. Therefore, it is still unclear what will be the outcome of using this product in region where highly virulent CSFV strain or CSFV with different degrees of virulence circulates. As additional features, the CP7_E2alf vaccine is not transmitted or shed through urine, feces, or semen [131] and the chimeric virus is genetically stable as has been revealed by both in vitro and in vivo studies [118,122,132]. 

The strategy of exchanging specific antigenic epitopes among different pestivirus species is another promising tool for the development of new CSFV marker vaccines. Thus, chimeric CSFV Riems variants expressing E2 genes with antigenic epitopes that were replaced with the respective epitopes from BDV Gifhorn were constructed [133]. The exchange of all three domains, A, B and C, resulted in a chimeric virus (vRiemsABC-Gif) that was able to induce protection against a virulent CSFV challenge, and the serological response of vaccinated pigs could be differentiated from that of infected pigs with commercially available E2 ELISAs. This candidate showed promising results as a potential live marker vaccine for oral and intramuscular immunization considering its effectiveness and its innocuousness [133]. 

Another chimeric pestivirus called CP7_E2gif has been reported as a noteworthy candidate, mainly if it is considered that it does not contain any gene from CSFV genome. Like the CP7_E2alf vaccine, CP7_E2gif contains the backbone from the strain CP7 of BVDV but the BVDV envelope protein E2 has been replaced by E2 from strain ‘‘Gifhorn’’ of border disease virus (BDV) [127]. The initial studies evidenced that the CP7_E2gif induced a strong immune response as soon as 10 days after vaccination with comparable levels to the vaccination using C-strain. An outstanding result was obtained when pigs vaccinated with CP7_E2gif developed protective immunity against challenge infection with CSFV Eystrup strain [134]. However, the fact that the specificity of CP7_E2gif induced antibodies will be directed against BVDV Erns and BDV E2, respectively, without a specific anti-CSFV capacity limits its use as vaccine. 

On the other hand, Flc-LOM-BE^rns^, another chimeric CSF live vaccine, was recently licensed by five Korean animal veterinary vaccine companies in 2016. This vaccine was developed by taking the infectious clone Flc-LOM, which is based on an attenuated live CSF vaccine virus (LOM strain), and removing the full-length classical swine fever virus (CSFV) Erns sequences and the 3′ end (52 base pairs) of the CSFV capsid. These regions were substituted with the full-length bovine viral diarrhea virus (BVDV) Erns gene sequence and the 3′ end (52 base pairs) of the BVDV capsid gene, yielding the Flc-LOM-BE^rns^ vaccine [128]. According to recent studies, this novel chimeric vaccine has shown to provide complete protection in pregnant sows [128,135]. The use of Flc-LOM-BE^rns^ in domestic pigs and bait vaccination of wild boar in South Korea has just started, hence the strength of the DIVA principle and the safety of this marker vaccine candidate is still unclear. 

We have discussed, so far, numerous CSF marker vaccine candidates, which have been designed based on sequences of closely related pestiviruses to CSFV [125,136,137,138]. However, the use of pestiviruses genetically and antigenically distant to CSFV has emerged as an attractive option, taking into consideration mainly the DIVA concept. Thus, three recent chimeric viruses have been developed by replacing the Erns of CSFV strain Alfort-Tübingen with the homologue gene of largely distant pestiviruses members [139]. The engineered chimeric viruses “Ra,” “Pro,” and “RaPro” contained Erns sequences from Norway rat and Pronghorn pestiviruses or a combination of both, respectively [139]. In this first study, all vaccine candidates conferred complete protection against clinical signs of CSF at 28 dpv. However, further evaluations of these candidates are required, in terms of effectiveness and safety and potential reversion to the virulence among others.

The identification of genetic determinants of virulence for CSFV have been an essential element allowing the scientific community with the use of a rational approach to design CSF live marker vaccines. The introduction of genetic mutations looking to attenuate one or more of these determinants into the CSFV genome using infectious clone can lead to the creation of safer and more efficient vaccines with DIVA properties. In this regard, Holinka et al. [140] reported the development of a live attenuated CSFV strain with two antigenic markers, called FlagT4v. A promising vaccine candidate marker was produced by introducing two separate genetic modifications into the backbone of CSFV Brescia strain [73,141]. The first is a synthetic epitope, Flag^®^ into the E1 gene, (Sigma, St. Louis, MO, USA) with the function of acting as a positive antigenic marker, and the second one is the mutation T4 (TSFNMDTLR) into the linear epitope TAVSPTTLR in E2 gene; this mutation disrupts the recognition of the epitope by the widely used monoclonal antibody WH303 [142], therefore, T4 acts as a negative antigenic marker [140]. During the vaccine assessment process, this new vaccine candidate was able to induce a full protection in swine since day 2 or 3 dpv depending on the route of administration (intramuscularly or intranasally, respectively). However, FlagT4v showed reversion to a virulent phenotype of wild-type CSFV [136] as evidences of genetic instability. In subsequent studies, the sequence analysis revealed deletions and substitutions almost exclusively in the areas of E1 and E2 where Flag and T4 were inserted [140]. To improve the genetic stability of FlagT4v, changes in the codon usage of these regions were introduced. The newly developed FlagT4Gv, showed to be stably attenuated when assessed in a reversion to virulence experiment was conducted, and conferred early effective protection against challenge with the virulent CSFV strain [136]. This promising vaccine has shown sterile immunity against challenge with the virulent parental virus beginning at 3 dpv, as well as increased levels of IFN-α in vaccinated animals [143]. The FlagT4Gv is, so far, one of the most promising vaccine candidates for emergency to use during an outbreak of CSF, however, DIVA tests relying on the positive and negative antigenic markers of this vaccine candidate are not available yet.

Just recently a new CSFV marker vaccine candidate called rHCLV-E2P122A has been reported [137] characterized by the presence of a single amino acid mutation into the ^122^PxA site of the epitope recognized by mAb HQ06,which was introduced using reverse genetic manipulation of the CSFV C-strain vaccine [137]. This new candidate vaccine was able to induce anti-CSFV neutralizing antibodies but not antibodies against the HQ06-recognized epitope in both rabbits and pigs, which can be differentiated from the antibodies induced by C-strain. However, is still unclear if the animals vaccinated with this engineered virus will be protected against the challenge with a wild viral CSFV-strain. 

## 5. Viral Vector and Replicon Vaccines against CSF

An additional approach pursued, since some years ago, has been the construction of trans-complemented CSFV deletion mutants, also known as CSFV-replicons [138,144]. Self-replicating RNAs (replicons) of positive strand RNA viruses are becoming powerful tools for gene expression in mammalian cells and for the development of novel antiviral and vaccines. Replicons are usually defined as replicative-competent viral genomes unable to generate infectious viral progeny due to a functional defect, caused by a partial or complete deletion of at least one structural gene [138,145]. Replicons can be packaged into the viral envelope to generate virus replicon particles (VRP) using a complementing cell line, which expresses the missing structural protein (trans-complementation) [138]. Notoriously, VRP can infect cells with the same efficiency as the parental virus, since their virion shell is indistinguishable from the envelope of the original virus. Thus, VRP fulfill one of the requirements for a safe vaccine, since they are non-transmissible either horizontally or vertically; based upon this, VRP do not produce an infectious progeny decreasing also the chances for the emergence of virulent virus from the vaccine. In addition, VRP have the advantage of prolonged antigen production to induce cytotoxic T-cell responses [145]. Furthermore, VRP do not require adjuvants as the replicative nature of the replicon generates RNA molecules that, through the Toll-receptors, trigger innate immune defenses providing the necessary signal to elicit the adaptive immune responses [144]. In addition, this type of vaccine allow differentiation of VRP-vaccinated from infected animals based on the absence of antibodies against the deleted protein(s) or epitope(s) in the VRP [24].

In the past, different CSF-VRP were developed with deletions of Erns, E2, or partial E2 sequences of C strain [146,147]. In all the cases, these CSFV replicons induced a protective response in pigs and were compatible with a DIVA approach to serology. However, differences in the level of protection of pigs against virulent CSFV were obtained depending of the replicon-type and the route of administration, for instance, the E2-deleted VRPs induced full protection after simultaneous intradermal, intramuscular, and intranasal injection [146], whereas the Erns-deleted VRP only showed protection after a parenteral immunization but not when the intranasal route was used [147]. 

Another CSF-VRP, deficient of Erns gene as a non-transmissible marker vaccine, were evaluated by Frey et al. [148]. In this regard, a cDNA clone of CSFV strain Alfort/187 was used. The vaccinated pigs with a single intradermal inoculation of VRP A187^Erns-^ elicited anti-E2 neutralizing antibodies, and a cellular immune response was determined by an increase in IFN-γ producing cells. Nevertheless, oral immunization resulted only in a partial protection, and the results obtained with the intradermal inoculation regarding the humoral and the cellular responses were not replicated [148].

As a general aspect, the CSF-VRP represent a robust and versatile system for gene expression and generation of vaccine to be used in pigs. However, it has been reported that the use of this type of vaccine is limited depending on the administration route. Nevertheless, a novel approach using adenovirus-delivery systems represent a promising alternative as it has been shown by the development of the candidate, Semliki Forest virus replicon-vectored marker vaccine “rAdV-SFV-E2.” rAdV-SFV-E2 is based on the replication-defective Ad5 vector that delivers a Semliki Forest virus replicon expressing the E2 gene of CSFV. rAdV-SFV-E2 has shown that it can elicit strong cellular and humoral responses in pigs, providing sterile immunity and complete protection against lethal challenge using virulent CSFV-strain comparable with the C-strain [149,150]. However, it is important to highlight that complete protection is achieved only after two doses of the vaccine [149]. Although further steps have been already taken in order to improve the immunogenicity of this candidate by a *Salmonella* enteritis bacterial ghost adjuvant, which can potentially improve the immune response of the host to the vaccine [151]. As an additional remark of this candidate, it was already observed that the efficacy of this vaccine was not interfered by the presence of non-related anti-CSFV antibodies and anti-BVDV antibodies or coadministration with live attenuated vaccines against other swine diseases [149,150]. Similarly, it was recently shown that maternally derived antibodies derived from the inoculation with rAdV-SFV-E2 were sufficient to provide some protection to piglets against lethal CSF challenge [152]. 

## 6. Discriminatory Tool for DIVA Vaccine

The success of a control program based on the application of a marker vaccine against CSFV relies upon an accurate discriminatory diagnostic test, which facilitates the differentiation between infected and vaccinated animals. The serological DIVA tests are focused on the discrimination between antibodies induced by natural infection of CSFV strains and the vaccine-derived antibodies [157]. This differentiation is based on the fact that during the development of the marker vaccines, antigens or epitopes were modified. One of the most commonly used companion diagnostic test of marker vaccines has been the strategy based on the detection of antibodies anti-Erns using an ELISA tool since the presence of antibodies anti-Erns are indicative of CSFV infection in pigs [86,89]. However, since specific antibodies against Erns are developed after 3–6 weeks post-infection, there is “blind-window” in the detection of infected animal mainly during the early stages of infection [158]. Likewise, the strategy of genetically engineered chimeric pestivirus has emerged as a promising option to generate efficient live marker vaccines, but the chimerization of closely related viruses keeps the problematic in terms of induction of cross-reactive antibodies [98]. For instance, the serological discriminatory test for the vaccine CP7_E2alf system is based on the detection of CSFV-specific Erns antibodies (for the presence of infected animals), since animals vaccinated will develop antibodies against CSFV-E2 but not against CSFV-Erns, while infected animals will develop antibodies against this last protein [117]. However, two major obstacles have been observed in the practical examples applied in the field, first, antibodies raised against BVDV Erns can cross-react with CSFV Erns, leading to false-positive results in animal vaccinated using CP7_E2alf, and as a second problem pigs infected with ruminant pestiviruses can also yield positive results when the discriminatory test is applied due to cross-reactivity or poor specificity of the test [159]. 

Currently, there are two CSFV Erns ELISAs commercially available, which have been evaluated as companion DIVA diagnostic tools for E2 subunit vaccines, such as CP7_E2alf, or similar chimeric vaccines. Thus, it can be found as PrioCHECK CSFV Erns commercialized by Thermo Fisher scientific (Thermo Fisher Scientific (former Prionics), Waltham, MA, USA), and as the pigtype CSFV Marker kit commercialized by QIAGEN Leipzig GmbH (Germany) [117]. The former have shown problems with the sensitivity of the test as well as cross-reactivity with other CSFV-related pestiviruses, including BVDV and BDV, which have limited its use [157]. It is important to highlight that several efforts have been made in order to optimize the performance of this assay [160,161,162]. However, PrioCHECK CSFV Erns (Thermo Fisher Scientific (former Prionics), USA) still exhibited deficiencies in terms of sensitivity and specificity, as well as robustness and reproducibility [115,117]. Meanwhile, the Erns-specific double-antigen ELISA (pigtype CSFV Erns Ab, QIAGEN Leipzig GmbH (Leipzig, Germany) has shown higher sensitivity and specificity parameters in combination with CP7_E2alf chimeric vaccine than PrioCHECK CSFV Erns (Thermo Fisher Scientific (former Prionics), USA). However, the effect of cross-reactivity with antibodies against ruminant pestiviruses was still observed [159]. Furthermore, a reduced specificity in both DIVA ELISA tests was observed when these discriminatory assays were applied on animals with maternal antibodies [163]. Hence, this aspect further complicates serological surveillance. In addition, recent studies have pointed out that the effect of multiple vaccination or the poor quality of the sample actually reduced the specificity of these ELISA tests leading to the conclusion that interpretation based on the application of these test can be only addressed under herd-based screening since the single sample interpretation is difficult to establish [37,164]. 

Interestingly, a novel companion DIVA ELISA for the marker vaccine rAdV-SFV-E2 has been developed using an enhanced expression system for the Erns protein [165]. Thus, Luo et al. [165] ropose a new indirect enzyme-linked immunosorbent assay (iELISA) based on the yeast-expressed Erns (yErns) using the methyltropic yeast *Pichia pastoris*. The results obtained from evaluating the new iELISA in a panel of swine sera revealed that the new assay had comparable sensitivity (94.6%) and specificity (97.1%) and showed consistence rates with the virus neutralization test. In addition, the iELISA showed higher sensitivity (90.4%) compared with PrioCHECK CSFV Erns (59.6%). Therefore, Luo et al. (2015) concluded that the yErns-based iELISA represents a promising DIVA test for E2-based marker vaccines against CSF [165]. 

It is evident that the future of the development of marker vaccine candidates will depend upon the successful development and implementation of a tailored DIVA assay. There is subsequent need to establish a formal link between the licensing of a new marker vaccine and the availability of a suitable DIVA assay to fulfil both requirements (protection and detection). In general, the development a DIVA assay to be used in combination with a marker vaccine faces two major challenges. The test must detect antibodies to CSFV with certainty and must not produce cross-reactivity with antibodies induced by ruminant pestiviruses. Therefore, the DIVA test based on the nucleic acid detection is the most promising strategies for an accurate and reliable differentiation of field virus infected from vaccinated domestic pigs and wild boars. In this regard, the strategy behind a genetic DIVA is based on the identification of genetic differences between vaccine strains and wild-type CSFVs. This approach is closer to be achieved if the exponential growing of the genetic databases is considered mainly if it is also taken into consideration that those animals under a chronic or persistent infection can excrete infectious viral particles for a lifetime period [166,167]. Thus, the use of highly sensitive molecular techniques has, nowadays, given rise to an increase in vaccine virus detections and differentiation [153,154,168]. In previous years, several research groups have developed some molecular biology-based CSF genetic DIVA systems. Real-time RT-PCR assay was developed by Leifer et al. (2009a) to specifically detect the C-strain for use as a genetic DIVA test in circumstances where this vaccine might be used. In the mentioned study, the differentiation between the strains was conducted by the detection of two nucleotide differences in the Erns encoding region of the viral genome at the 3’ end of each primer [155,156]. A one-step RT-PCR using TaqMan minor-groove-binding probes was also developed to distinguish between attenuated Korean LOM and wild-type strains of CSFV in Korea [169]. Another differential assay has been developed that can distinguish the genetically similar Riems C-strain and HCLV and LPC vaccine strains from most field strain genotypes except some of the genotype 3 strains [170]. In addition, a differential real-time RT-PCR assays specifically designed to detect the individual challenge strains were developed [160]. On the other hand, the current emergence of isothermal amplification assays including loop-mediated isothermal amplification system (LAMP), which have been already applied for the diagnostic of CSFV [19], or recombinase polymerase amplification (RPA) have opened new opportunities to the development of DIVA assays. This type of diagnostic tool has several advantages in comparison with the thermal amplification systems, including they can be applied nearest at the point of care, have higher level of sensitivity and specificity, are cost effective, and are less impacted by the sample degradation-effect process [171]. These approaches combined with the novel detection methods using cas13a [161] and cas12 [162] represent the future of the DIVA assays. 

## 7. Emergence of New CSFV Subgenotype in Endemic Regions under Vaccination Programs

After the second half of the last century, countless efforts have been addressed by many government authorities to control and eradicate CSF from national pig populations, however, the disease remains as a significant challenge for the scientific community and one of the most important diseases for animal health [16] During several decades, live attenuated vaccines, mainly C-strain vaccines, have been used in the implementation of those control programs against CSF in endemic countries [22,39,72]. Reasons that support the extensive use of this type of vaccine are mainly based on their high efficacy, safety as well as they are considered cost-effective products [21,27]. Several studies accomplished by different research groups have revealed that the efficacy of live attenuated vaccines can be impaired by multiple factors, including the presence of maternal antibody [22,29,51], low viral loads in the inoculum [21], breaks into the cold-chain (storage or transportation), route of vaccination, and age of the animal [22] as well as the co-infection with immunosuppressive pathogens [28,31,172,173,174]. These issues can indeed lead to vaccination failures contributing to sporadic outbreaks and reemergence of CSF in vaccinated herds, which in the practice have shown to be a real concern since several strains with the capacity to escape to the immune response of the host can emerge [16,17,175,176].

The previous statement gains significance if it is considered that the genetic variability of CSFV is relatively high [177,178] compared with other RNA viruses for instance infectious bursal disease virus [179,180]. However, unlike other RNA viruses, the genetic diversity of CSFV is not driven either by mechanisms of reassortment or by homologous recombination. In this last aspect, only one strain has been found as a recombinant (CSFV GenBank Ass. No. AF407339) [181], but other research groups have been unable to replicate this result [176,182,183], suggesting that the mechanisms of recombination likely do not influence the genetic diversity in CSFV. These elements evidence that the accumulation of point mutations seems to play the main role as a genetic mechanism driving the variability of CSFV. However, accumulated evidences from different environments including endemic regions with zones without vaccination [184], where the competence effect take place, or countries under inefficient vaccination programs, where positive selective pressure are in place [17,175,185], revealed that those variable can also impact the viral fitness differently. Indeed, contrary at what it is expected that in endemic region without a vaccination policy where all potential variant can be expressed results indicated a genetic stability in the viral population [184,186], whereas changes in the genetic composition, virulence and pathogenicity have been found in circulating strains from endemic regions under inefficient vaccination programs [16]. These results obtained from molecular epidemiology studies support the idea that bottleneck effects generated by the lack of sterilizing immunity by conventional vaccines has contributed to emergence of new CSFV-strains [17,175,176,187]. Thus, these variants that escaped to the immunity induced by the vaccine can favor the viral persistence in the swine population with changes in the clinical manifestation of the disease toward the generation of chronic and persistent forms of CSF [16,17,20,46,176,188]. Therefore, as a major aim of the current review, it is relevant to critically illustrate those examples where changes in the genetic composition, antigenic composition, or virulence of the circulating CSFV-strain have taken place caused by a long-lasting inefficient vaccination program.

### 7.1. Cuba

The first records of CSF in Cuba date from the 1940s due to the importation of infected pigs from the United States [189]. In 1965, the lapinized C strain was imported into the country and a national production of a vaccine started by a commercial entity (LABIOFAM, S.A.), which was considered a critical step to the control program of the CS. Indeed, CSF-outbreak was not reported in Cuba since mid-1970s suggesting a status of CSF-free. However, in 1993, despite the vaccination program implemented, CSF reemerged in the country [190]. A subsequent study based on molecular epidemiology approaches indicated that the CSF-reemergence was caused by the reintroduction to the field of a highly virulent strain, “Margarita,” isolated in 1958 and used as a challenge strain in the vaccine potency tests since 1965 [191]. Due to the continuous occurrence of outbreaks, in 2002, CSF was declared endemic [192]. Here, after 10 years of reemergence, a tendency towards milder forms of the disease, characteristic of the chronic disease presentations in the infected animals, was observed [190]. In fact, Diaz de Arce et al. (2005) reported the presence of high rate of non-synonymous mutations in the CSFV partial E2 gene suggesting that the evolution of the virus in the country could be linked to the trend towards mild disease presentations [189]. Likewise, Pérez et al. [17] reported about the inability of the vaccine in use to confer a sterile immunity causing a “bottleneck” effect and acting as a positive selection pressure that facilitates the emergence of a viral subpopulation with changes in virulence and pathogenicity of the strains, which emerged as escaping mutants to the neutralizing antibodies induced by the vaccine [17]. A recent study also confirmed that the action of the positive selection pressure induces a decrease in virulence, with alterations in pathogenicity and antigenicity without causing major genetic variability [16]. It was also suggested by a study performed in parallel that the circulation of these new viral escaping variants under condition of partial immunity induced by the vaccine favored the viral persistence in the swine population with presence of either prenatal or postnatal infections in piggles as a result of a failure in the response to the vaccination in sows [20].

Epidemiological approaches recently performed shed lights about the occurrence of CSF in the Cuba, suggesting that in this country, a slight trend to increase the incidence of the disease over the years as a results of the endemism conditions together with the lack of a successful implementation of a control policy has enabled the viral circulation [193]. 

Indeed, the CSF endemic conditions in Cuba have been linked to the broad circulation of positively selected strains in the field, characterized by moderate and low virulence [20,192], which have favored the presentation of subacute and chronic forms of the disease leading to the persistence of viruses in the pig populations. An additional concern to this complex epidemiological situation is the fact that the primary diagnosis of CSFV in Cuba is based on immunohistochemistry [19]. This method is prone to false negative since those samples containing low viral low are mainly tested negative, hence this aspect also contributes to the current underreport of CSF in this country facilitating the circulation of the virus in the pig herds. Due to the lack of success in the previous control program, the Cuban Veterinarian authorities are currently implementing additional control measures to improve the current situation regarding CSF. These measures include, the introduction of a new eradication plan implemented by zones [194], with the use of novel *in-house* developed vaccine (Porvac vaccine). This new policy is being assessed in the country with promising results.

### 7.2. Ecuador

CSF was firstly reported in Ecuador in the 1940s [7]. In 1978, the government started recording the occurrence of the disease as part of the epidemiological surveillance system, which was coordinated by the national animal health program [7]. However, the presence of CSF in Ecuador was officially registered in the OIE records from 2006, when three outbreaks were reported, and the disease has been present in the country thereafter (OIE, 2013). Since 2011, the National Program for the CSF Control and Eradication was implemented in Ecuador using a CSFV attenuated C-strain vaccines [195]. In the past, through molecular epidemiology studies, it was revealed that Ecuadorian strains were located into the CSFV subgenotype 1.1 clade [175]. However, recently, the emergence of the new subgenotype 1.7 in Ecuador was reported [177]. Despite the causes that originated this event have not been disclosed yet, studies to uncover if these novel strains emerged as consequence of the positive selection pressure caused by the escape to the vaccine are in progress.

### 7.3. Brazil

In Brazil, the first report of CSF was as early as 1888, in the states of Minas Gerais and São Paulo [176], but it was until 1992 that an eradication program was implemented in this country [182]. Since Brazil has such a vast territory, the eradication of the CSF in the whole federation in a single step was considered neither technically nor economically feasible, hence, the program was designed to achieve the status of CSF-free in a progressive way, starting on those areas where the swine production was more intensive [196]. After 6 years of efforts based on the implemented program, a non-vaccination regimen was introduced into the biggest pig production regions, while it was kept on other regions of the country [197]. The national recognition of the first free zone took place in 2001 [196] and currently, Brazil has an status of CSF-free zones that comprises approximately 82% of the national pig herds, and a non-free zone, located on the North and Northeast regions of the country, with approximately 18% of the national pig herd. However, many CSF outbreaks still occurred in the CSF non-free zones despite the adoption of health measures addressed to eliminate the occurrence of the diseases. In fact, the disease has been causing significant social and economic impacts and concerns about the possible reintroduction of CSFV in the Brazilian free-zone during the last few years [198]. Examples of these concerns resulted indeed in the emergency of two new subgenotypes [8] suggesting that the evolution of the virus in the field is leading to a broad genetic diversity in this country. Therefore, in 2019, a new plan entitled, Brazilian CSF-Free Strategic Plan, was created with the aim to eradicate the infection from the CSF non-free zone, accompanied by strict sanitary guidelines [198]. 

### 7.4. China

The first report of CSF in China was in 1925 [183]. Since the HCLV strain vaccine was developed in 1954, the vaccination with this vaccine strain has been implemented for more than 50 years as part of the control program [199]. However, sporadic CSF-outbreaks still occur and the sows with persistent CSFV infection has been identified as the main source for the viral transmission to suckling and weaned piglets [185]. Moreover, several reports denoted a change in the virulence of the CSFV strains with presentations of the diseases from the acute form to a subacute and chronic manifestations [187,188,200,201,202,203]. Thus, several studies also concurred that the prolonged vaccination program, without a successful implementation in the field, could have caused those changes in the pathogenicity and antigenicity of the new emerging strains [175,187,204] favoring the high prevalence of chronic cases of CSF in this country. Hence, a complex situation is currently present in China in regards to CSF where the disease is considered endemic [185,187].

Since 1990s four different CSFV-subgenotypes have been circulating in China (1.1, 2.1, 2.2, and 2.3), with the subgenotypes 2.1 and 2.2 considered the predominant ones [183,205]. However, in recent decades, there has been a swift and the subgenotypes 2.3 and 2.2 became silent, while subgenotype 2.1 has emerged with a predominant role in the outbreaks all across the country [206]. Previous studies have shown that this specific subgenotype (2.1) rapidly evolves generating a considerable high genetic diversity with 10 clades denoted as subgenotypes (2.1a-2.1j) [203,207]. However, a detailed analysis accomplished in Rios et al. (2017) showed that neither the genetic divergence showed by the lineages nor the statistical values in the topology resolved were enough to support the classification of these lineages as new subgenotypes [178]. Nonetheless, it is important to highlight that these same authors proposed to define the new CSFV Subgenotypes 2.4 and 2.5, previously defined as Subgenotypes 2.1d and 2.1c by Gong et al. 2016 [177], as an indicative that the genetic diversity of CSFV circulating in China has increased in the recent years. An interesting evidence, which highlights the high level of genetic diversity of CSFV circulating in China, is the case of the Chinese strain S171 isolated from commercial fetal bovine serum batches [208]. This CSFV-strain is genetically distant from the previously reported CSFV genotypes, which has been found circulating in cattle, and it has been recently suggested as a new outgroup into the CSFV specie [209]. Likewise, other CSFV isolates have been found circulating in cattle in this country as evidence of spillover from pigs [210]. However, if all these new emergent strains are a direct consequence of the inefficient vaccination, it needs to be addressed in future studies.

### 7.5. India

The first case of CSF in India was reported in 1944, localized at the Northern parts with the subsequent expansion to other regions of the country [211,212]. Since then, CSF has been considered endemic in India and the disease has been under a control program based on an approach of massive vaccination [213]. Since 1964 a lapinized vaccine containing the CSFV-*Weybridge* strain included into the subgenotype 1.1 has been used as part of this control program. Despite the control program, a high prevalence of CSF has been reported in most of the states of the country [214] with the circulation of several subgenotypes including 1.1, 2.1, and 2.2 across the country. Similar to other CSF-endemic regions discussed in this current work, India is experiencing a switch from the previous dominant subgenotype 1.1 to more recent genotypes such as 2.1 and 2.2 [215,216,217]. However, a recent study defined as new subgenotype 2.4 to CSFV strains that circulated in India during 2012–2013 previously located in genotype 2.1 [177], but so far, the subgenotype 2.2 has been determined as the current dominant [217]. 

In order to improve their current control program, the Indian Veterinary Research Institute have incorporated a new safe, live attenuated CSFV vaccine using local strain. This vaccine has been claimed as the best choice for use in the CSF Control Program in the country [218]. Notoriously, like the findings reported in China, studies based on molecular epidemiology revealed the presence of the CSF genome in cattle serum samples. The sequencing of the full length E2 revealed the similarity to the CSFV 2.1 genotype. As a remarkable feature was described by this authors that the bovine samples that tested positive were mostly from farms that were in close proximity to pig farms, suggesting spillover from pigs [219], which represent a more complex situation for the control of the disease in India.

### 7.6. Vietnam

CSF is endemic disease in Vietnam and live attenuated CSFV vaccine (HCLV) have been used as a national program to control and prevent the spread of CSFV [204,220]. Similar to other regions under vaccination programs, some outbreaks of CSFV frequently occur in Vietnam, where three different CSFV-genotypes have been identified [221]. In 1991, a CSFV strain belonging to subgenotype 1.1 (VN91) was isolated in Hung Yen province, which is similar to the genotype strain commonly used as CSF vaccine in Vietnam [204]. However, the CSFV strains circulating in Vietnam during the 2014 outbreak, with moderate virulence [222], were reported as subgenotype 2.1 [223], evidencing the emergence of a new subgenotype in the country despite the control program implemented. However, a recent study proposed a new CSFV classification, consisting of five main genotypes (1–5), with seven subgenotypes in each of genotype 1 and 2 [177]. In this new scheme for genotyping CSFV, the Vietnamese CSFV strains classified as 2.1 were reclassified as subgenotype 2.5 and 2.6 [177]. Recently, subunit vaccine “VN91-E2” have been developed in Vietnam, for CSF prevention and control in the endemic area and emergency outbreaks of CSF in this country [221].

### 7.7. Russia

In Russia, CSF is considered an endemic disease. The control program for CSF is based on extensive vaccination with the CSFV LK VNIIVVIM live vaccine carried out for more than 50 years [224]. However, cases in wild boar and outbreaks in domestic pigs have been often reported. All Russian CSFV strains from the late 1990s clustered within subgenotype 1.1 [224]. Interestingly, in a recent study, the outbreaks among 2007–2014 predominantly genotypes 2.1 and 2.3 have been reported [225]. The recent genotype 2.3 isolates from Russia are phylogenetically closely related to the isolates from Latvia and isolates that had caused CSF outbreaks in Central Europe [5]. Within the CSFV genotype 2.1, virus isolates from Russia phylogenetically group together with isolates from China and other Asian countries [225]. Despite the wide application of the vaccine and the implementation of sanitary measures, the disease is far from being controlled, but an increase in genetic diversity of the virus with different clinical manifestation has raised instead. 

### 7.8. South Korea

The first report of a CSF outbreak in South Korea dates back to 1908 [226]. Since then, sporadic outbreaks have been reported throughout the nation and CSF has been recognized as one of the most devastating diseases that continuously cause great economic losses to the swine industry [44]. Live attenuated LOM vaccine have been widely used across the country to eradicate CSFV in the field since 1974, and it has been proven to be safe and highly immunogenic in pigs for many decades [42]. The South Korean government established a plan for CSF eradication in June 1996 [42]. As result of this eradication program, Jeju Island became a CSFV–free area, and vaccination efforts ceased there in 1999. Meanwhile, a no-vaccination policy in the mainland of South Korea in 2001 was implemented. However, the disease reemerged in 2002 in the mainland of South Korea with many outbreaks and the vaccination was started again [44]. Despite the mandatory vaccination policy ruled for several years, sporadic CSF outbreaks have occurred in mainland South Korea [42,43,44] suggesting either an evolutionary process of circulating strains toward an escaping lineage of CSFV or a potential reversion to virulence by the vaccine strain. In addition, it has been informed that since 2002, CSF in Korea has undergone an antigenic shift from genotype 3 to genotype 2 [227]. In 2014, CSF reemerged in naïve pig herds on Jeju Island, South Korea, caused by the reversion to the virulence by the LOM vaccine strain [46]. The LOM virus has spread in the herd population and caused clinical signs in young pigs and pregnant sows. At this time, this region considered as CSF-free during decades has become endemic to CSFV [47,48]. Very recently, vaccination with the new Flc-LOM-BErns started in South Korea, as part of the CSF control program. 

### 7.9. Japan

In Japan, the first outbreak of CSF was reported in 1888, since then, many outbreaks occurred repeatedly for over 100 years [182]. In 1969, a CSF attenuated live vaccine (GPE- vaccine) was developed in Japan and was applied nation-wide in the field in the same year. Since then, the outbreaks of CSF drastically decreased, with the last outbreak occurring in 1992. The eradication program was started in 1996 and in 2007, Japan was given CSF-free status from World Organization for Animal Health. In September 2018, CSF reemerged in Gifu Prefecture, Japan, for the first time in 26 years, affecting domestic pigs and wild boars [228]. The causative virus is closely related to isolates in East Asia and is classified under subgenotype 2.1 [229]. Due to the fact that many, new notifications of CSF cases in both wild boar and domestic pigs were being reported continually, the government decided to apply the routine administration of a bait vaccine to wild boars in March 2019 and the preventive vaccination in domestic pigs in the affected prefectures in October 2019 to constrain further CSF spread [76]. However, despite strenuous control efforts, almost 2 year after the initial CSF notification, the lack of success in controlling the outbreak is concerning. The continuous notification of CSF in the area might have been attributed to wide spread of the virus within wild boar populations favored by free animal movements, as well as to the emergence of epidemiologically related domestic pig farms [76,230].

## 8. Future Remarks

The *status quo* regarding CSF in several countries with the aggravated condition linked to the emergence of new CSFV genotypes in endemic regions caused by systematic inefficient vaccination [20,121,222], it is a clear indicative that there is an urgent need to improve the control programs currently in place. There is also a need of more efficient vaccines against CSFV with the ability to confer sterilizing immunity, hence, this aspect deserves high priority for the research community. 

Currently, the immunoinformatic, as an emerging scientific discipline, has had an improvement in scientific research in the prediction and characterization of epitopes with high precision [231]. The evaluation in silico of CTL epitopes provides the possibility to design vaccines with more and wider protection in comparison with conventional vaccines. Thus, the use of this tool could provide a better information about a possible escape from emerging strains of CSFV to vaccines used to control the disease, mainly in endemic areas [232]. To prevent many diseases in the future, the epitope-based vaccines have shown to be an excellent candidate strategy. In comparison with traditional vaccines, epitope-based vaccines have many unparalleled advantages including low cost, multivalence, no genetic component, efficient antigen presentation, ease of application as well as the absence of infectious potential. Indeed, multi-epitopes vaccines have been successfully developed against other viruses of wide genetic variability such as dengue virus [233], Ebola virus [234], chikungunya virus [235], and hepatitis C virus [236]. Nevertheless, those candidates have not been applied in the practice yet. Recently, ours research group conducted for the first time, the characterization of epitopes of B-cell and CTL-cell of CSFV strain with the combination of immunoinformatic and classical techniques to analyze metric distances of antigens between ancestral and emerging CSFV strains. This type of study facilitates the understanding on how new emerging CSFV strains could escape to both cellular and humoral responses induced by the vaccine used to control the disease, mainly in endemic areas [16]. This combination of immunoinformatic with classical immunology, can facilitate the development of antigenic maps for CSFV and contribute to the application of successful control measures, mainly for emerging strains of this viral agent. In this regard, it is undeniable that the advantages that the characterizations of epitopes can provide to establish a platform for the development and application of multi-epitope vaccines against CSFV. This type of vaccines will allow to cover a broad range of variability of epitopes from different strains, including those that could escape to the current vaccines in use or to other vaccines in the future. Therefore, this is an area that will require from different research groups time, effort, and resource for further investigations. 

## Figures and Tables

**Table 1:** Summary of the vaccine and vaccine candidates against classical swine fever virus (CSFV) discussed in the current work.

Vaccine and Vaccine Candidates	Type of Vaccine	Essentials Characteristics	References
**LOM vaccine**	Live attenuated vaccine	Potential reversion to a virulent strain.	[39,40,41]
**C-strain**	Live attenuated vaccine	Safe and effective against the disease. Protective immune response against all CSFV genotypes. Early protection in the absence of neutralizing antibodies.	[24,36,153,154]
**GPE-strain**	Live attenuated vaccine	Safe in pregnant sows, newborns, and adult pigs. Early protective immunity. Potential reversion to a virulent strain.	[63,65,66]
**Thiverval strain**	Live attenuated vaccine	Safe and genetically stable. Prevent vertical transmission.	[62]
**PAV-250**	Live attenuated vaccine	Safe in pregnant sows and genetic stable. Elicit a strong immunogenic response. Protect against different virulent strains of CSFV.	[63,64,65]
**INFα-E2-CSFV**	Subunit vaccine	Recombinant human alpha interferon has an immune-stimulatory effect. Clinical protection 7 days after single vaccination. Double vaccination with a 3-week interval/challenge six weeks after booster vaccination with highly virulent CSFV. Duration of immunity is of at least 9 months after double vaccination.	[117,120,155,156]
**KNB-E2**	Subunit vaccine	Sterilizing protection after a single vaccination dose. Protection against CSFV strain two weeks after double vaccination with 3-week interval. Protection against challenge is 32 weeks after double vaccination.	[83,94]
**TWJ-E2^®^**	Subunit vaccine	Full protection against a challenge using the CSFV highly virulent strain after two doses.	[81,84]
**E2-CD154**	Subunit vaccine	CD154 as a molecular adjuvant enhancing the immune response. Full protection against the challenge of virulent strains of CSFV as earlier as 7 dpv after a single vaccine dose and in the absence of protective neutralizing antibodies. Prevent against the vertical transmission of the very virulent CSFV strain in pregnant sows. Stable for at least 24 months at room temperature.	[105,106,113]
**Suvaxyn^®^ CSF Marker**	Live marker vaccine (chimeric)	Clinical and virological protection after the challenge against virulent and moderate CSFV strain. Complete protections after a week of applying a single intramuscular dose, and the immune protection for at least 6 months. Genetically stable. Prevent against the vertical transmission of the moderate virulent CSFV strain in pregnant sows.	[125,128,129,131,140]
**vRiemsABC-Gif**	Live marker vaccine (chimeric)	Protection against a virulent CSFV challenge after intramuscular vaccination, partial protection after oral immunization.	[133]
**CP7_E2gif**	Live marker vaccine (chimeric)	Induced partial protection with no transmission after challenge with CSFV virulent strain 28 days post vaccination. Safety and marker properties	[127,134]
**Flc-LOM-BErns**	Live marker vaccine (chimeric)	Provide complete protection in pregnant sows.	[128,135]
**FlagT4Gv**	Live marker vaccine (two antigenic markers)	Full protection in swine since day 2 or 3 dpv depending on the route of administration (intramuscularly or intranasally, respectively). Provide sterile immunity against challenge with the virulent parental virus beginning at 3 dpv, as well as increased levels of IFN-α in vaccinated animals	[115,148,149]
**“Ra,” “Pro,” and “RaPro”**	Live marker vaccine (chimeric)	Vaccine candidates conferred complete protection against clinical signs of CSF at 28 dpv.	
**rHCLV-E2P122A**	Live marker vaccine	Marker properties	[137]
**VRP A187delE(rns)**	Replicon vaccine	Protection at 23 days post vaccination against challenge with a single intradermal inoculation elicited anti-E2 neutralizing antibodies and cellular immune responses determined by an increase in IFN-γ producing cells.	[148]
**rAdV-SFV-E2**	Replicon vaccine	Elicit strong cellular and humoral responses in pigs. Providing sterile immunity and complete protection against lethal challenge using virulent CSFV strain 7 weeks after double vaccination. Protection through maternally derived antibodies. Enhancement through *Salmonella* ghost adjuvant.	[149,150,151,152]

## Data Availability

No new data were created or analyzed in this study. Data sharing is not applicable to this article.
